# Primary Cutaneous Anaplastic Large Cell Lymphoma Arising from Lymphomatoid Papulosis, Responding to Low Dose Methotrexate

**DOI:** 10.4103/0974-2077.58525

**Published:** 2009

**Authors:** AS Nandini, Venkatram Mysore, S Sacchidanand, Suresh Chandra

**Affiliations:** *Venkat Charmalaya, Centre for Advanced Dermatology, Bangalore, India*; 1 *Department of Dermatology, Bangalore Medical College and Research Centre and Registrar, Rajeev Gandhi University for Health Sciences, Bangalore, India*; 2 *Manipal Hospital, Bangalore, India*

**Keywords:** Anaplastic large cell lymphoma, cutaneous lymphoproliferative disorders, lymphomatoid papulosis, methotrexate

## Abstract

CD30+ cutaneous lymphoproliferative disorders (CLPDs) present variable clinical and histological manifestations. We report here a case of an adult male patient who progressed from lymphomatoid papulosis to anaplastic large cell lymphoma. The patient responded satisfactorily to a low dose of methotrexate.

## INTRODUCTION

The spectrum of CD30+ lymphoproliferative disorders of the skin includes CD30+ cutaneous anaplastic large cell lymphoma (ALCL), lymphomatoid papulosis (LyP), and other miscellaneous entities.[1] Clinically, both LyP and ALCL present with varied manifestations, leading to difficulty in diagnosis. LyP has been known to progress to ALCL[2,3] and its diagnosis needs immunomarker studies. The case reported here is notable for its initial presentation with spontaneously remitting lesions, followed later, however, by the lesions becoming persistent. CD30 stain confirmed the diagnosis and the lesions resolved satisfactorily with low dose methotrexate.

## CASE REPORT

A 52 years-old male patient presented with multiple, asymptomatic lesions of 2 years' duration all over the body. These lesions were solid, elevated, red in color, and of varying sizes. They had initially started over the extremities and had gradually progressed to involve the chest, back, and abdomen. The lesions tended to heal spontaneously over a period of 15 to 20 days but, would recur at same or different places. The patient also had a history of blood-stained, watery discharge from a few lesions on the right arm and the left leg. The left leg lesion had ulcerated which had led to an initial clinical diagnosis of pyoderma gangrenosum. A skin biopsy, at that time, was reported as showing a predominantly diffuse, mixed, inflammatory infiltrate without any specific diagnosis. The patient had been treated with dapsone and oral steroids.

Two months before the visit, patient had noticed a change in the behavior of lesions. There was a fresh crop of firm lesions over the back that was persistent. There was no history of weight loss, generalized weakness, bleeding tendency, fever or recurrent infections and the patient was not a known diabetic or hypertensive.

**Figure 1:**
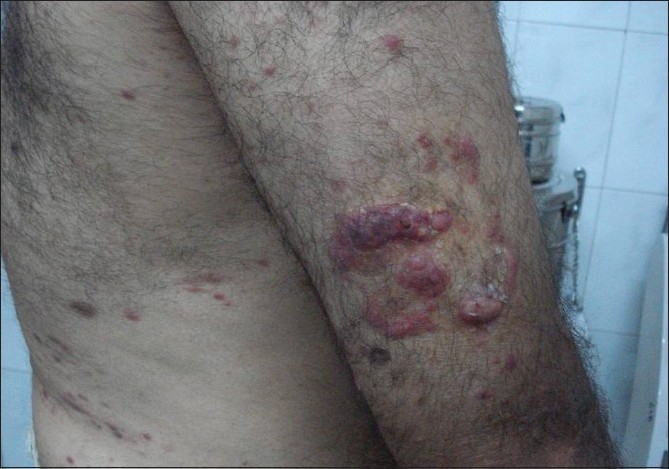
Erythematous papules, plaques, and nodules in generalized distribution

**Figure 2:**
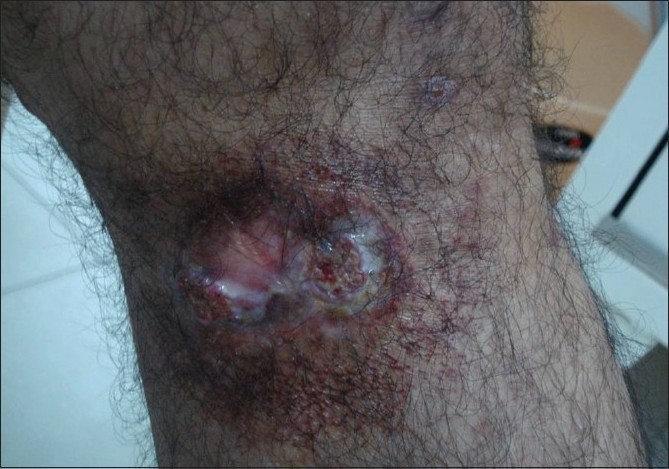
Ulceration of plaque seen

**Figure 3:**
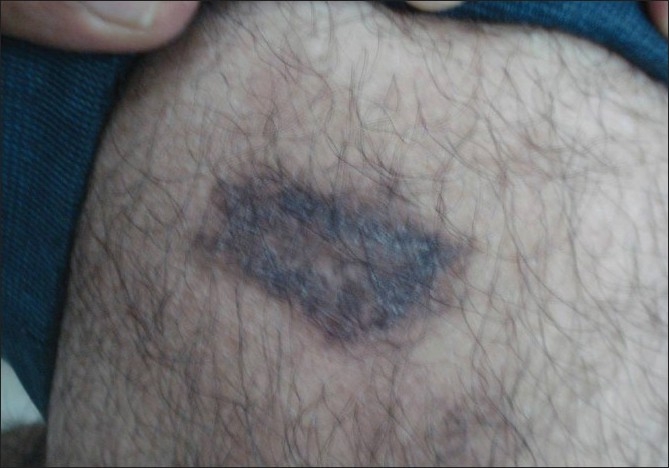
Resolved lesion with hyperpigmentation

**Figure 4:**
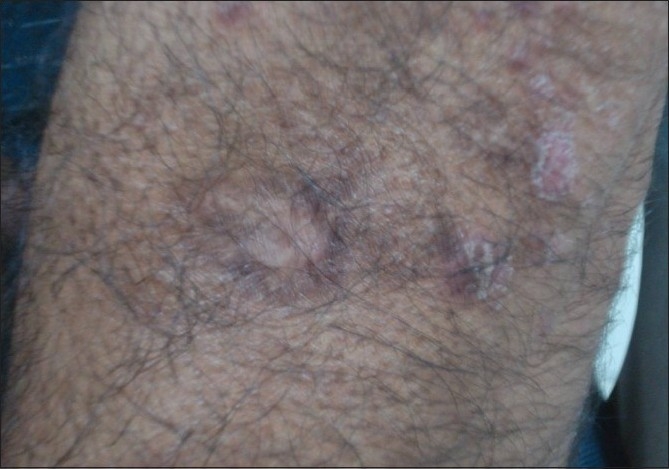
Resolved lesion with scarring

Clinical examination revealed lesions distributed all over the body, but predominantly over the extremities, back, chest, and abdomen [Figure 1]. The palms, soles, face, scalp, oral cavity, and genitalia were spared. The lesions consisted of red to red-brown papules, nodules, plaques, and ulcerated plaques [Figure 2] with crusting. There were many resolved lesions on the extremities that showed hyperpigmentation and scarring [Figures 3 and 4]. Systemic physical examination results were not remarkable.

The previous skin biopsy slides when reviewed by a dermatopathologist (the second author of this paper) showed a mixed cellular infiltrate in the upper and mid dermis with extravasation of RBCs and focal epidermal necrosis. The infiltrate consisted of polymorphonuclear cells, lymphocytes, plasma cells, and occasional atypical cells with large nuclei. Correlating the histological features with the clinical picture at this stage, provisional differential diagnoses of lymphomatoid papulosis and cutaneous T cell lymphoma were considered.

**Figure 5:**
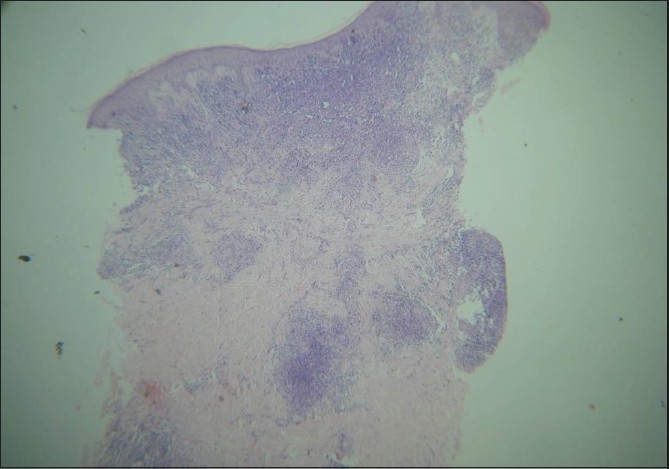
Dense mononuclear infiltrate throughout dermis extending into subcutis (Stain H and E ×25)

**Figure 6:**
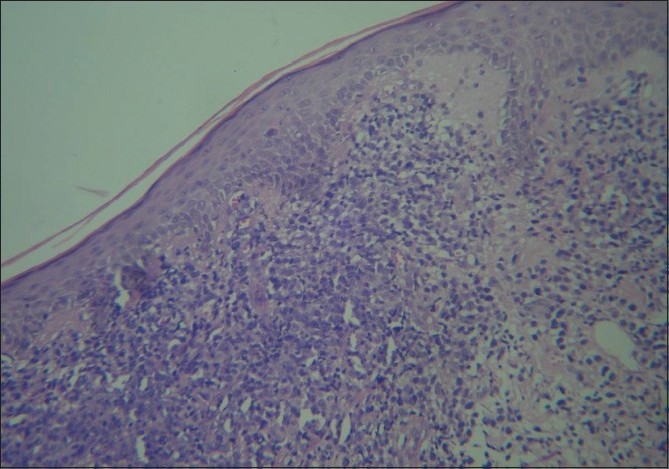
Many large lymphocytes with atypical nuclei seen (Stain H and E ×100)

**Figure 7:**
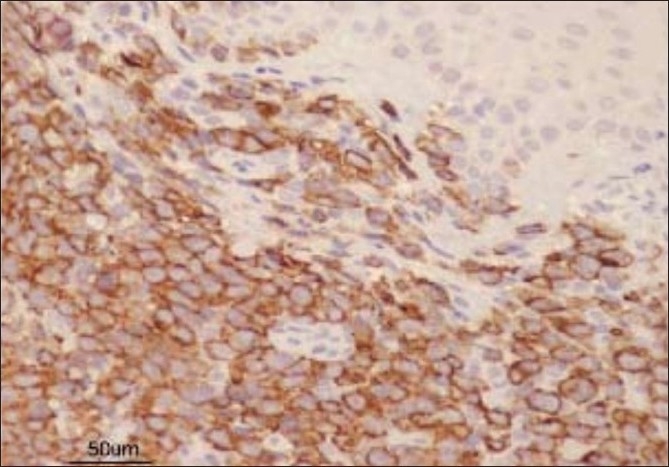
Immunonohistochemistry showing CD30-positive cells

**Figure 8:**
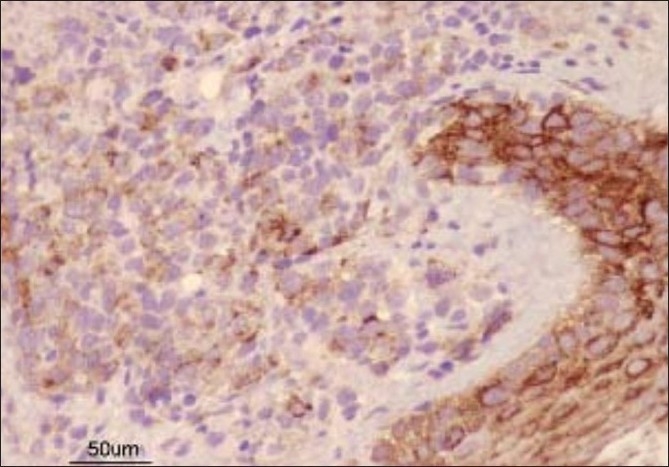
Immunohistochemistry showing EMA-negative cells

Fresh skin biopsies were performed from multiple sites and showed dense, monomorphous, mononuclear infiltrate throughout the dermis, extending into the subcutis [Figure 5]. Many cells were large (20 microns) and had large atypical nuclei [Figure 6]. Atypical mitotic figures were seen; the epidermis was normal. Immunohistochemical analysis revealed that cells expressed CD30 [Figure 7] and were negative for EMA [Figure 8]. Background lymphocytes expressed CD3 and CD20, features that were consistent with anaplastic large cell Lymphoma, possibly of T cell lineage.

A complete hemogram including peripheral smear, biochemical and serological tests showed no abnormalities. A chest X-ray and CT scan of the abdomen, chest, and pelvis showed no systemic or nodal involvement. The absence of any systemic lesions confirmed the diagnosis of primary cutaneous anaplastic large cell lymphoma without systemic involvement.

**Figure 9:**
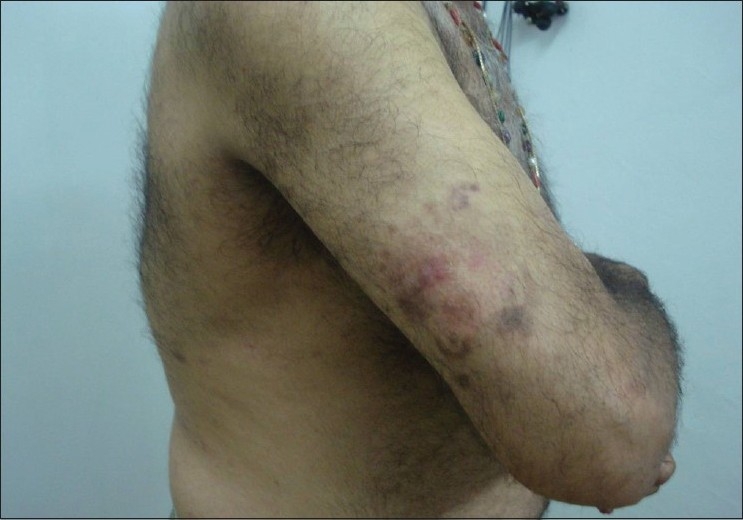
After treatment (after eight months of treatment)

Our patient was started on a low dose (7.5 mg/week) of methotrexate. He responded dramatically and the lesions subsided completely within two months of initiating treatment [Figure 9]. He has been under follow-up for over nine months without any relapse.

## DISCUSSION

This case presents interesting chronological changes in morphology and histology. The earlier clinical picture of recurrent, self-healing lesions lasting for two years, and the initial histological picture of mixed infiltrate, large atypical cells, extravasated RBCS, all suggest a diagnosis of lymphomatoid papulosis (LyP) for this stage of the disease process. The subsequent change in behavior of lesions occurring two months ago to indolent and persistent lesions and dense, monomorphic infiltrate with large atypical cells staining positively with CD30, suggests a transformation to CD30+ anaplastic lymphoma. The absence of systemic lesions confirmed its primary cutaneous onset. Thus, the final diagnosis of the patient was CD30 positive primary cutaneous anaplastic large cell lymphoma (PCALCL) arising in previous LyP.

The group CD30+ cutaneous lymphoproliferative disorders (CLPDs) includes a spectrum of disorders such as lymphomatoid papulosis, borderline cases of CD30+ CLPDs, and primary cutaneous anaplastic large cell lymphoma (PCALCL).[1] These entities constitute the second most common group of cutaneous lymphomas, after mycosis fungoides, according to the newly revised WHO and EORTC Consensus classification.[1] Clinically, both lymphomatoid papulosis and ALCL present with variable manifestations and therefore, cause difficulty in diagnosis. Furthermore, LyP has been known to progress to ALCL[3] so that the distinction of LyP from PCALCL may not be possible in all cases. The initial diagnosis is often difficult and lesions may be confused for various inflammatory conditions, as in our case.[4]

Primary cutaneous anaplastic large cell lymphoma is a rare anaplastic CD30+ large *T*-cell lymphoma originating and usually confined to the skin, characterized by solitary or locoregional occurrence of reddish or brownish nodules and tumors, with a tendency to ulcerate.[2] Progression to extracutaneous sites is rare, but has been reported in about 10% of the cases.[5] PCALCL has a favourable prognosis with a disease-related survival rate of around 90%.[5]

'Lymphomatoid papulosis' describes a 'self-healing rhythmical paradoxical papular eruption histologically appearing "malignant", but having a relatively benign clinical behaviour'.[5] The primary lesion typically is an erythematous, papule or nodule that spontaneously regresses over few weeks with scaling, crusting, ulceration, and possible scarring with atrophy, hyperpigmentation, or both. Lesions often present as crops or generalized eruptions. Overall, the clinical course resolves over a span of 10-20 years although frequent relapses are the norm. It has a benign clinical course with a 10-year survival rate of nearly 100%.[5,6] Some (10-20%) of the cases are associated with malignant lymphoma including mycosis fungoides, PCALCL, and Hodgkin's disease.[6]

Histologically, LyP and ALCL may be difficult to distinguish, and clinicopathological correlation is often necessary to establish a diagnosis.[6] This was indeed so in our case.

Therapies shown to be effective in the treatment of LyP include excision, topical corticosteroids, topical mechlorethamine, oral antibiotics, phototherapy, low-dose methotrexate, interferon α, and systemic retinoids including bexarotene.[6] Treatment of PCALCL should always be tailored to the extent and severity of cutaneous involvement. Systemic agents may be appropriate for generalized PCALCL, and the main categories of therapies include chemotherapy and biological therapies such as interferon or oral bexarotene.[6] Only in patients with extracutaneous disease or systemic lymphoma is systemic chemotherapy (like CHOP regimen) indicated. For localized disease, excision (if the extent of disease is limited) and local radiation (for greater tumor burden) are the two most common forms of treatment. Several local treatments have been reported to be successful in patients with limited disease. These include a 308nm excimer laser in the case of a solitary cutaneous CD30+ lymphoproliferative nodule[7] and intralesional methotrexate in a case of PCALCL.[8] The nodules within the plaque were injected with a total of 0.5mL of methotrexate (25 mg/mL) followed by one additional treatment of 0.4mL of methotrexate (25mg/mL) a week later. The treated area had dramatically flattened one week after the second treatment.[8] A case report discusses two patients with non-regressing primary cutaneous CD30+ *T*-cell lymphoma that was successfully treated with topical imiquimod 5% cream (Aldara, 3M) three times weekly for six weeks.[9]

Low-dose methotrexate has also been advocated in patients with disease limited to the skin.[3] In our case, low-dose methotrexate with 7.5 mg weekly oral administration has proved to be successful in inducing remission, without any adverse effects.

## CONCLUSION

Our case is notable for the progression of LyP to ALCL. The morphology and the progression of lesions were found to change with time. The case is also notable for the good response to a simple treatment with methotrexate.

The case underscores the need for proper clinico-pathological correlation, the importance of immuno-histochemistry, and the need for regular follow-up and repeated biopsies for proper diagnosis and management.
